# (Semi-)Automatically Parsing Private Protocols for In-Vehicle ECU Communications

**DOI:** 10.3390/e23111495

**Published:** 2021-11-11

**Authors:** Tongtong Chen, Xiangxue Li

**Affiliations:** 1 School of Software Engineering, East China Normal University, Shanghai 200062, China; 51174506053@stu.ecnu.edu.cn; 2 Shanghai Key Laboratory of Trustworthy Computing, Shanghai 200062, China; 3 Westone Cryptologic Research Center, Beijing 100070, China

**Keywords:** CAN, ECU, in-vehicle network, private protocols

## Abstract

In-vehicle electronic control unit (ECU) communications generally count on private protocols (defined by the manufacturers) under controller area network (CAN) specifications. Parsing the private protocols for a particular vehicle model would be of great significance in testing the vehicle’s resistance to various attacks, as well as in designing efficient intrusion detection and prevention systems (IDPS) for the vehicle. This paper proposes a suite of methods for parsing ECU private protocols on in-vehicle CAN network. These methods include an algorithm for parsing discrete variables (encoded in a discrete manner, e.g., gear state), an algorithm for parsing continuous variables (encoded in a continuous manner, e.g., vehicle speed), and a parsing method based on upper-layer protocols (e.g., OBD and UDS). Extensive verifications have been performed on five different brands of automobiles (including an electric vehicle) to demonstrate the universality and the correctness of these parsing algorithms. Some parsing tips and experiences are also presented. Our continuous-variables parsing algorithm could run in a semi-automatic manner and the parsing algorithm from upper-layer protocols could execute in a completely automatic manner. One might view the results obtained by our parsing algorithms as an important indicator of penetration testing on in-vehicle CAN network.

## 1. Introduction

Modern vehicles become more and more networked and intelligent, which not only brings passengers better driving experiences, but also introduces more attack surfaces. Folklore attack surfaces include universal serial bus (USB), Bluetooth, Wi-Fi, cellular network, and other communication interfaces, as well as software vulnerabilities of in-vehicle operating systems [1,2,3,4,5,6]. For these attack surfaces, there exist complete penetration testing methods to making practical exploit [7]. For in-vehicle electronic control unit (ECU)—the essential components on CAN bus however, there does not exist any mature methods of penetration testing.

In-vehicle security. Crucial information, such as diagnostic, informative, and controlling data, is transmitted through the CAN bus to implement various vehicle services, such as autonomous driving and assisted driving [8,9,10,11]. Since communication security was not a primary concern at the beginning of CAN design, it is not a surprise that the in-vehicle network is exposed to numerous security threats [12,13,14,15,16]. For example, any ECU can hear from any other ECU on the CAN bus without the capability of identifying the real sender’s identity. This may easily lead to attacks (e.g., packet injection and data manipulation) and risk the safety of the passengers. The community has seen extensive IDS on in-vehicle network [17,18,19,20,21].

Attack surfaces of ECUs. In-vehicle ECUs have two main attack surfaces, i.e., the attack by injecting original CAN message and the replay attack by unified diagnostic services (UDS). One might perform ECU penetration testing as below: inject CAN messages to check the fault tolerance of ECUs, parse the private protocols of the original CAN messages, bypass UDS security authentication, and verify the operability of reprogramming ECU, etc. Therein, one essential step is to parse private protocols of the original CAN messages. Since CAN messages do not participate in the interactions of upper-layer protocols and there is no security restriction related to upper-layer protocols, the attackers could first understand the relevant control principle and then inject power-related CAN messages to achieve the control of steering or braking. Thus one might view the number of parsed private protocols as an important indicator of penetration testing.

Parsing private protocols. CAN bus is widely equipped in modern vehicles and CAN specifications are regulated as open standards [22]. However, the contents of IDENTIFIER and DATA FIELD contained in CAN frames are privately defined. Each vehicle manufacturer has its own specifications. Private protocols of CAN messages might be parsed by black-box means (parsing and verification) or gray-box means (ECU firmware reverse) [23,24,25]. There exist few detailed mechanisms on parsing CAN private protocols in the literature. Miller and Valasek sketched their methods for parsing CAN private protocols and the corresponding CAN message injection techniques [26,27,28], but they did not illustrate the details of parsing steps for any given vehicle.

Main contributions. This paper looks into the traffic captured on the CAN bus and proposes a suite of methods to parsing private protocols for in-vehicle ECU communications. These methods are universally applicable to most vehicle models on the market. The parsing results are of significance in testing the vehicle’s resistance to various attacks, as well as in designing efficient intrusion detection/prevention systems (IDPS) for the vehicle. For example, the results can assist the subsequent firmware reverse task. These methods also provide substantial pre-processing for scientific research, e.g.,  vehicle physical variables for abnormal driving detection [29] and in-vehicle intrusion detection [30,31]. We present the following parsing methods:

(1) An algorithm for parsing discrete variables. It requires two pieces of CAN traffic (say traffic A and traffic B). The  state of the variable to be parsed should remain unchanged in traffic A, and change alternately in traffic B and one of the alternate states should be the same as that in traffic A;

(2) An algorithm for parsing continuous variables. It performs multiple filtering operations, each of which requires two pieces of CAN traffic. Filtering operations should specify the trend (increasing, decreasing, or unchanged) of the variable to be parsed in the two pieces of CAN traffic, discard those variables that do not meet the trend and further obtain the expected one that invariably conforms to the trend. We can run this algorithm in a semi-automatic manner;

(3) A parsing method based on upper-layer protocols. Upper-layer protocols, such as OBD and UDS, can be used to obtain the current value of certain physical variable of a vehicle. These values are used as a reference, and the values of the original data on the CAN bus are fitted to obtain the content to be parsed. We can run this method in an automatic manner.

Roadmap. The remainder of this paper is organized as follows. Section 2 reviews CAN frames and UDS protocol. Section 3 and Section 4 present the algorithms for parsing discrete/continuous variables, respectively. The parsing method based on upper-layer protocols is presented in Section 5. Section 6 performs experimental analysis to illustrate the correctness and the efficiency of these parsing algorithms. Section 7 introduces some tips for practical applications. Conclusions are given in Section 8.

## 2. CAN Data Frame and UDS Protocol

**Figure 1:**
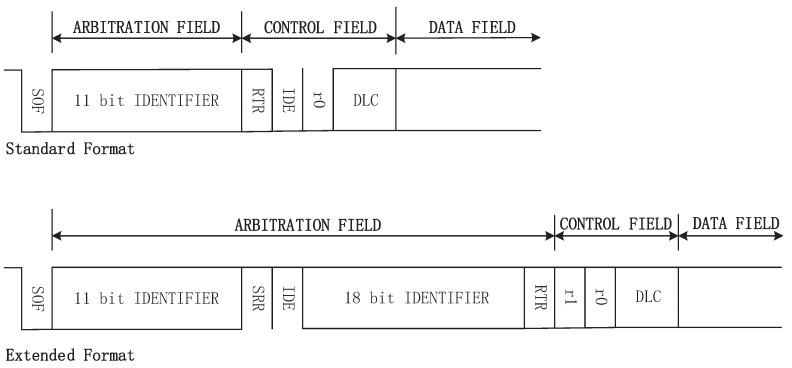
Data frame.

There are four types of CAN frames: DATA FRAME, REMOTE FRAME, ERROR FRAME, and OVERLOAD FRAME. This paper just considers DATA FRAME as the last three types are unusual at vehicles’ normal states. A DATA FRAME is composed of seven different fields: START OF FRAME, ARBITRATION FIELD, CONTROL FIELD, DATA FIELD, CRC FIELD, ACK FIELD, and END OF FRAME. As shown in Figure 1, there are two types for DATA FRAMEs: standard and extended formats. A standard/extended frame has 11-bit/29-bit IDENTIFIER, respectively. The difference between the two frame formats is distinguished by the IDE bit in CONTROL FIELD. This can be readily processed by dedicated CAN transceiver chip. Thus our parsing algorithms focus on IDENTIFIER in ARBITRATION FIELD, DATA LENGTH CODE in CONTROL FIELD, and DATA FIELD.

Defined in ISO 14229-1 standard [32], UDS is a diagnostic communication protocol provided for in-vehicle ECUs, with the format of Service Identifier, Sub-function, and Parameter. The OBD protocol also conforms to this format. The mapping of the UDS protocol on CAN is divided into single frame, first frame, consecutive frame, and flow control frame, as defined in ISO 15765-2 standard [33]. Table 1 shows the specific definition of each frame. The single frame is responsible for carrying messages with data length of no more than 7 bytes. The first frame and consecutive frame are for messages of length greater than 7 bytes, and the last one is for flow control.

## 3. Parsing Discrete Variables

Given a vehicle, its discrete variables include gear state, wiper action indication, and window lifting state, etc. We decouple the process of parsing discrete variables to two stages. Take light on/off state as an example. The first stage is to collect the CAN traffic with the requirement that the turn signal states remain unchanged. Thus, those data with changed states could be removed. In the second stage, we turn on and off the turn signal alternately, collect CAN traffic during this period, and continue to remove those data that do not meet the number of above alternation operations. Now the remaining contents indicate the states of the turn signal.

We use code fragment 1 to elaborate our design trick. It specifies the data structures used in the discrete-variables parsing Algorithm 1. For description convenience, it takes a linear table, and more efficient data structures might be adopted to improve filtering efficiency. The listDiscrResult linear table stores the final filtering results grouped by IDENTIFIER and its size is equal to the number of different IDENTIFIERs that appear in the collected CAN traffic. The nodes stored in listDiscrResult follow DiscrParsing structure, where id member is IDENTIFIER, and len member is DATA LENGTH CODE. listStep1/listStep2 stores all values that appear in the first/second stage for each filtering unit, respectively. listCount stores the number of times each filtering unit generates alternations at the second stage (based on the first stage). The filtering unit determines the lengths of listStep1, listStep2, and listCount. As DATA LENGTH CODE of a CAN frame is up to 8 bytes, the length of the related table is 8 if the filtering unit is 1 byte, and  16 if the filtering unit is a nibble. It is not easy to observe the filtering results quickly if one sets the filtering unit too large or too small. Practically, a nibble is used as the filtering unit and we can achieve high filtering efficiency by jointly exploiting other parameters such as UnchangedElementSize. UnchangedElementSize/ChangedElementSize upper-bounds the number of different values that occur for the filtering unit in the 1st/2nd stage, respectively. AlternationCount lower-bounds the times each filtering unit generates alternations in the second stage.
**Algorithm 1** Data structure of discrete-variables parsing algorithm.uint UnchangedElementSize;uint ChangedElementSize;uint AlternationCount;struct DiscrParsing {      uint id;      uint len;      List<List<byte>> listStep1;      List<List<byte>> listStep2;      List<List<uint>> listCount;};List<struct DiscrParsing> listDiscrResult;

We illustrate the filtering operation of the first stage below. Using the CAN traffic collected in the first stage, we update each listStep1 member in listDiscrResult table with IDENTIFIER being primary key. If the number of different values in a filtering unit for listStep1 member is greater than UnchangedElementSize, the corresponding index is set as invalid. Namely, the filtering unit for this IDENTIFIER has been removed, and the subsequent filtering comparison will not be performed on this filter unit.

Then, the filtering operation of the second stage follows. Using the CAN traffic collected in the second stage, we update each listStep2 member in listDiscrResult table with IDENTIFIER being primary key. If the value of each filter unit is not in listStep1, it will be recorded in listStep2. If the number of different values in a filtering unit for listStep2 member is greater than ChangedElementSize, then the corresponding indexes in listStep1 and listStep2 are set as invalid. In the meanwhile, we record the times the values of each filtering unit alternate. By generating an alternation we mean that the value of the current filtering unit and the value of the last corresponding filtering unit are not synchronously in listStep1 or listStep2. Take the turn light on/off state as an example and set the off state ‘off’ in listStep1 and the on state ‘on’ in listStep2. Then, there are two alternations in the ‘off off off on on on on off off’ state sequence. If the number of the alternations for a filter unit is less than AlternationCount, the  indexes corresponding to listStep1 and listStep2 are set as invalid.

After three filtering operations in two stages, the final filtering results could be obtained by traversing the filtering units for the listStep1 member in each node of listDiscrResult.

## 4. Parsing Continuous Variables

Given a vehicle, its continuous variables include tire angle, engine speed, vehicle speed, and most physical variables related to power. The data structure of continuous-variables parsing algorithm is similar to that in prior section. It also uses IDENTIFIER as grouping label to filter each unit. 1/2/0.5/1.5 bytes are common filtering units for continuous-variables parsing algorithm. The  index of filtering unit is based on ‘sliding’ combination. Take 2-byte filtering unit and big endian byte order as an example: for a CAN data frame with DATA FIELD 0 × 01, 0 × 02, 0 × 03, and 0 × 04, the corresponding values of each filtering unit are 0 × 0102, 0 × 0203 and 0 × 0304.

**Figure 2:**
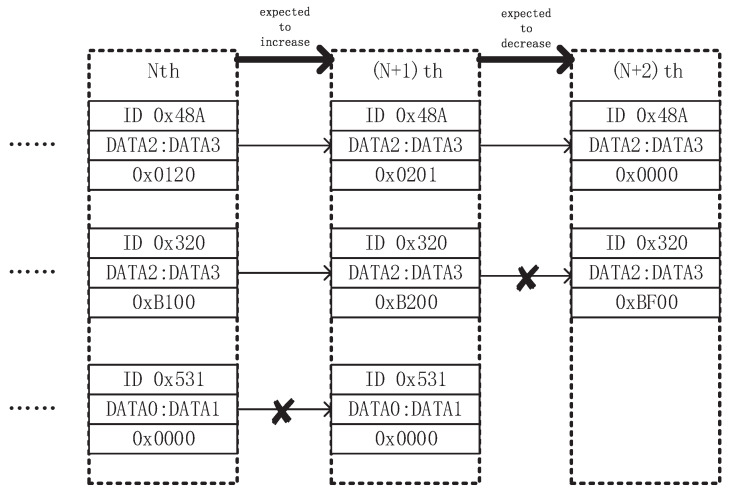
Continuous-variables parsing process.

Compared with discrete-variables parsing algorithm, continuous-variables parsing is more heuristic. We use Figure 2 to elaborate our design trick. We obtain ‘snapshot’ of data frames with different IDENTIFIERs in two different states. Therein, ‘snapshot’ refers to the data frame with the same IDENTIFIER that only retains the latest time when data frames are received. After obtaining the ‘snapshot’ of the data frames with different IDENTIFIERs in two different states, we specify the changing trend (increasing, decreasing, or unchanged) of the filtering unit to be parsed in these two states, and remove the filtering units that do not meet the conditions. The filtering step would be repeated several times until desirable results occur.

## 5. Parsing Method Based on Upper-Layer Protocols

It seems hard to analyze physical variables that are unperceivable or lack relevant cognition, e.g., engine torque. There also exist deceptive cases. For example, if two physical variables (say throttle percentage and engine speed) are correlated and have the same trend of change, then we can not distinguish them well. To handle this kind of obstacle, we introduce upper-layer protocols (e.g., OBD, UDS) to make automatic parsing. OBD protocol contains many physical variables related to emissions. ReadDataByIdentifier and other services in UDS protocol can obtain more information of physical variables. We also group the parsing process by the IDENTIFIER of CAN frame. The parsing unit is the same as that in Section 4 and common units include 1/2/0.5/1.5 bytes. The parsing unit uses a ‘sliding’ combination and is related to the byte endian. We fall back on code fragment 2 to elaborate our design trick. It specifies the data structure used in the Algorithm 2. CANFrame is a basic DATA FRAME structure containing IDENTIFIER, DATA LENGTH CODE, and DATA FIELD. The baseY member in the MultiPoints structure is the value of current physical variable parsed by the upper-layer protocol at some moment. Each node in the rawXs table is a CAN frame with a different IDENTIFIER that appears for the first time before and after the time at which baseY is obtained. listPoints is a collection of coordinate points referenced by the value obtained by the upper-layer protocol. The length of the listPoints table is equal to the number of times the request service is sent. The length of the rawXs table in the MultiPoints structure is equal to the number of different IDENTIFIERs.
**Algorithm 2** Data structure of parsing method from upper-layer protocol.struct CANFrame {      uint id;      uint len;      List<byte> data;};struct MultiPoints {      uint baseY;      List<struct CANFrame> rawXs;};List<struct MultiPoints> listPoints;

We send the upper-layer request instruction at a reasonable time interval, and simultaneously record the corresponding CAN traffic. The returned data frame of the upper-layer protocol are sequentially positioned in the received CAN traffic, and parsed (according to the frame structure introduced in Section 2) to obtain a value indicating current state. baseY is obtained at this point. We add CAN data frames with different IDENTIFIERs of the first occurrence before and after the frame containing this baseY to the rawXs table, and thus obtain a complete MultiPoints node. After the CAN traffic is processed, listPoints stores the (x,y) coordinate set corresponding to each parsing unit of each different IDENTIFIERs. listPoints can be traversed to take (x,y) coordinates as a set of samples. Then, we fit the sample data of each group. According to the common sensor signal processing methods and the definition of data scaling in documents, such as ISO 14229-1 and SAE J1939, the data transmitted on CAN bus are much more likely to form a linear relationship with the actual physical variables. In the actual scenario, a linear fitting method can be used to perform a good fitting, or a more advanced fitting model can be used for further parsing.

We take as example the linear regression of two-dimensional data (which also enjoys easy implementation), and the least-square method could be used to obtain the fitting result. Let the fitting line be

(1) Y=kX+b

Then, we obtain *k* from the sample data and *b* from undetermined coefficients.

(2) $$k=\frac{\bar{xy}-\bar{x}\cdot \bar{y}}{\bar{x^{2}}-{\bar{x}}^{2}}$$

Once obtaining the fitting line for each parsing unit of different IDENTIFIERs, we calculate the standard deviation between the true value and the fitting value of each group and take the group with the smallest standard deviation as the final parsing result.

**Figure 3:**
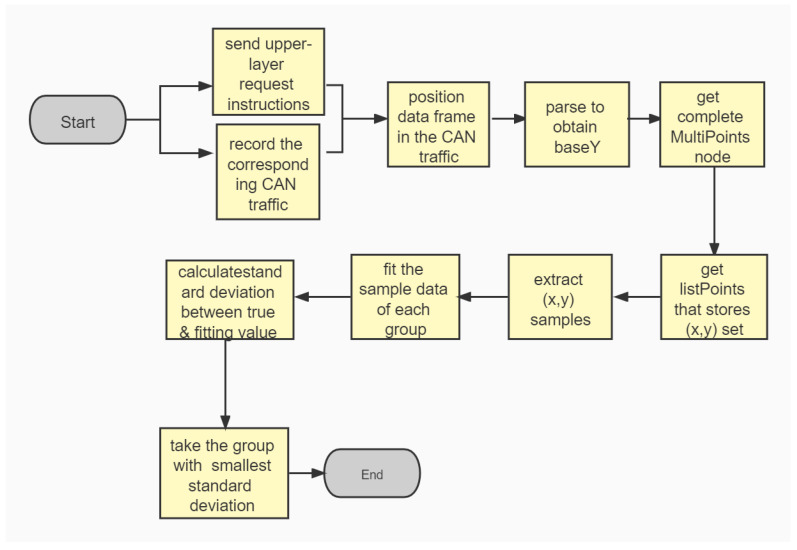
Parsing method based on upper-layer protocols.

Figure 3 gives the flowchart of our parsing method based on upper-layer protocol.

**Figure 4:**
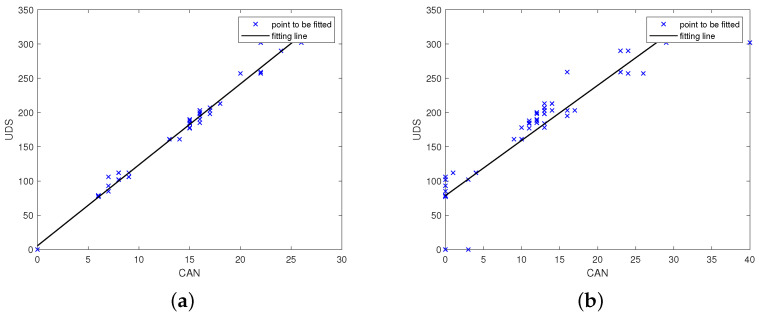
Parsing results of engine torque. (**a**) minimum standard deviation. (**b**) second small standard deviation.

Figure 4a,b show the minimum standard deviation (standard deviation: 17.33) and the second smallest group (standard deviation: 40.96) in the output of parsing the engine torque. The parsing units corresponding to the two subfigures are the 4th byte of the DATA FIELD of IDENTIFIER 0 × 280 and the 4th byte of the DATA FIELD of the IDENTIFIER 0 × 488. It is known from subsequent experiments that the physical variables represented by the 4th byte in the DATA FIELD of the IDENTIFIER 0 × 488 is the accelerator pedal position. Note that the engine torque will be larger as the throttle is stepped on. The results meet this intuition.

**Figure 5:**
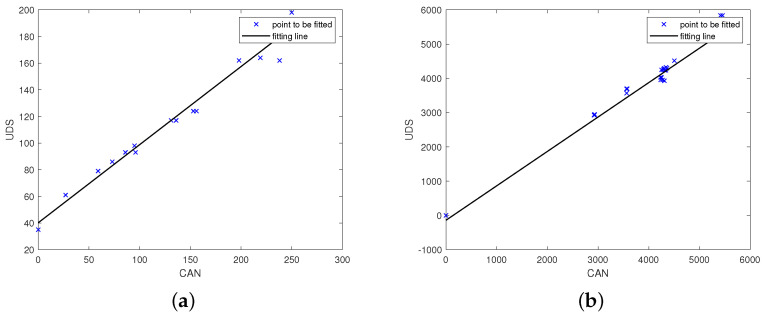
Parsing results of relevant physical variables. (**a**) accelerator pedal position. (**b**) engine speed.

As shown in Figure 5a, when the value returned by upper-layer protocol indicates the accelerator pedal position, the standard deviation of the 4th byte in the DATA FIELD of the IDENTIFIER 0x488 is the smallest. There exist strong correlations among engine speed, engine torque and accelerator pedal position and we can parse these variables under practical conditions. As can be seen from Figure 5b, the engine speed on the CAN bus of the experimental car is the same as the value returned by the UDS.

## 6. Experimental Analysis

Now we illustrate the correctness and the efficiency of the parsing algorithms. The experiments are conducted on five different brands of vehicles, including an electric vehicle. Three parsing algorithms can all be implemented on these five vehicles. Although the electric vehicle does not support the OBD protocol, it does support the UDS protocol. Note that all algorithms execute with low costs and our experiments are performed on a laptop (core i5 and RAM 8 G).

### 6.1. Correctness

Both discrete- and continuous-variables parsing algorithms are based on the filtering trick, and the variables to be parsed might not be filtered out as they satisfy the filtering conditions, which guarantee that the contents after filtering operations must include the variables needed to be parsed. Filtering conditions are set in the algorithm to remove the contents that do not meet the conditions, and the contents left contains the desired variables to be parsed.

The correctness of parsing algorithms can be verified from various dimensions. For most variables related to the comfort function and the dashboard, one could inject the parsed results onto the CAN bus and observe the subsequent effects. For example, CAN frames containing the variables related to wiper scraping might be injected to check whether the wipers scrape or not. In case that only one parsing unit is included in the final parsing result, one need further confirmation of the parsing unit. Take the filtering results of high-light brightness for example, one could toggle the light control button to check whether the parsing unit is in line with the actual situation. The parsing method from upper-layer protocols might be useful in solving the problem that the final parsing results contain more than one parsing unit. This kind of parsing variables are generally power-related and have strong correlations. Thus, we may not verify them by simple injection and the correctness of these variables would not be strictly proved by black-box method alone. One possible trick is to further validate these values by firmware reverse parsing. The contents of firmware reverse parsing are linked with power-related calibration data and CAN upper-layer protocols. We use multiple fitting points in various states to argue that the fitting results are rational. As shown in Table 2, 0 means that the accelerator pedal is not pressed, and 1 for fully pressed. The table gives the parsing unit with the smallest standard deviation and its corresponding standard deviation. The experiments use different number of fitting points and consider all positions of the accelerator pedal. The parsing results are consistent.

### 6.2. Efficiency

Next, we discuss the efficiency of the parsing algorithms. These algorithms have no strict requirements on time complexity. As explained in Section 3, Section 4 and Section 5, each of them only takes linear time to process the traffic frame by frame. Moreover, the lengths of storage tables used in the algorithms are small as the minimum filtering unit is 1 bit, the number of different IDENTIFIERs of CAN frames on one CAN bus does not exceed 100, and 100 fitting points suffice for parsing.

**Figure 6:**
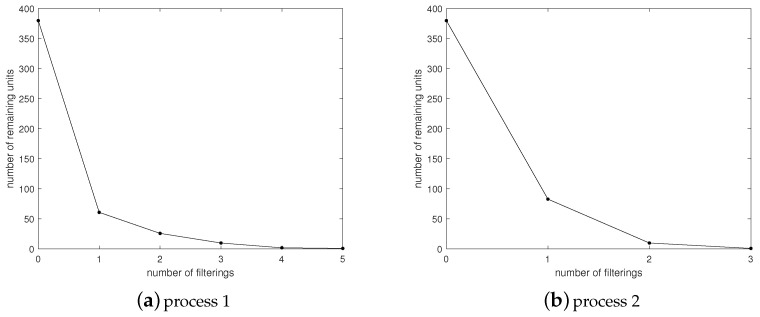
Filtering process.

The efficiency of the filtering operations depends on the filtering conditions. In practice, continuous-variables parsing algorithm in Section 4 is less efficient than discrete-variables parsing in Section 3. This is partially due to the fact that the number of filtering results is not stable. The quality of the comparative samples determines the quality of the filtering. Figure 6a shows the process of parsing the brake booster pressure at the point that the vehicle starts but the engine does not start. We then change the brake pressure by stepping on the brake and compare and filter the CAN traffic under different pressures. A total of five filtering operations are performed. As shown in Figure 6b, the last filtering operation collects the CAN traffic in the engine startup state, using only three filterings in total. Generally speaking, the continuous-variables parsing algorithm provides satisfactory efficiency, and can analyze one content in at most two minutes by collecting 15 s of CAN traffic each time.

The discrete-variables parsing algorithm in Section 3 uses only two pieces of CAN traffic samples. By collecting 15 s of CAN traffic each time, a content can be parsed in at most one minute. For five cars in our experiments, we have the accuracy of 100%. Table 3 shows the percentage of parsing units removed by the three filtering conditions of the algorithm in parsing the left turn signal (the engine is not started). The first stage removes a small number of unrelated parsing units. We have most data unchanged when the relevant operations are not performed on the dashboard. The second stage is mainly to prevent the omission of certain parsing units with timing characteristics, which are unchanged in the first stage but changed in the second stage. There are no such units in this filtering process. The condition of alternating times removes most of the parsing units that do not satisfy the condition, and this primarily contributes to the efficiency of the algorithm.

The method from upper-layer protocols in Section 5 could provide automatic parsing. This can be completed by setting up the upper-layer protocol instructions to be sent and driving the vehicle for a short period of time to collect the power data in different states. As shown in Table 2, as long as the variables to be parsed give changeable values, the parsing results will not be affected (even if there are fewer fitting points). If upper-layer protocol instructions are sent every 2 s and 20 fitting points are collected, then only 40 s suffice to analyze relevant content.

## 7. Discussions

### 7.1. Correlation of Protocol Encoding

One may note that there exist tight relationships between some variables, such as left doors and right doors, front lights and back lights, etc. It is natural that the manufacturers define similar protocols for their similar functionalities. This might help us parse the protocols with reduced efforts (rather than double costs). In the process of parsing, we could take into account the correlation of protocol encoding to significantly reduce parsing cost.

For example, if a datum unit with a certain IDENTIFIER is parsed to indicate the left turn signal, then there is a high probability that the right turn signal is also with this IDENTIFIER. As another example, if we already parse that the left door related state is represented by 2 bytes and presented in certain manner, then it is much likely to that relevant state corresponding to the right door is also represented by 2 bytes and presented in the same manner.

### 7.2. Targeted Fuzzy Testing

Generally speaking, fuzzy testing is a common method of private protocol analysis. In spite of its effectiveness, it is not an efficient option. However, this would be much time-consuming in the case of no response information. In fact, one has to enumerate all possible values for a given variable. As an extreme case, there are $2^{64}$ possibilities one has to try for the 8-byte data field of a particular identifier. On average, one can find the correct protocol in $2^{63}$ tries.

By the correlation of the protocol encoding, our parsing methods might be used to locate certain variables quickly and the subsequent targeted fuzzy testing can be performed on the CAN frame corresponding to the parsed variables. This will really reduce parsing cost. Consider the parsing of dashboard related protocols. We can locate the IDENTIFIER associated with the dashboard. As the dashboard has a poor ability to handle conflicts, we could obtain good feedback when injecting data frames related to the dashboard. One can identify more private protocols quickly by performing a fuzzing testing on the frames corresponding to this IDENTIFIER.

### 7.3. Full View on Physical Variables

It is important for the researchers to capture a full view on the in-vehicle variables, e.g.: how many variables might be defined in the vehicle, what are their functionalities, are they co-related (if yes, what is the relation), etc. This is meaningful, either in launching an effective attack on the vehicle or designing an efficient IDPS. It would make no sense if the variables to be parsed are not encoded in the original CAN frames. It is thus meaningful to obtain a full view on physical variables provided on the vehicles. After parsing private protocols of five different vehicles, we confirm that physical variables can be divided into three types, i.e., vehicle comfort functions, dashboard, and power.

Physical variables associated with comfort functions include the wiper, the door, the window, and rear view mirror, etc. Injecting operations on this kind of private protocols generally receive positive feedback. Thus, we can inject relevant CAN frames to control wiper watering/scraping, unlock/lock doors, and fold mirrors, etc.

Private protocols with respect to the dashboard mainly include light control and pointer control on the dashboard. The injection of frames related to the dashboard will be responsive and all the dashboard information described in the vehicle manual might be parsed, e.g., turn signal, profile light, airbag light, speed pointer, speed pointer, oil level pointer, and so on. Additionally, some alarms might be obtained in the meanwhile, e.g., overspeed alarm, oil leakage alarm, fuel temperature alarm, etc.

Physical variables with respect to vehicle power mainly include vehicle speed, engine speed, throttle opening, accelerator pedal position, fuel pressure, standard load value, brake pedal position, brake booster pressure, body angle, tire acceleration, gear position, air conditioning status, etc. This kind of injection operations generally require the knowledge of ECU firmwares and relevant processing logic to make the injection frames work.

## 8. Conclusions

This paper introduces three methods of parsing ECU private protocols on in-vehicle CAN network. Each method has appropriate application scenarios, i.e., discrete/continuous variables and physical variables related to vehicle power. We demonstrate the correctness and the efficiency of the algorithms. Some parsing tips and experiences are also presented. Our continuous-variables parsing algorithm can run in a semi-automatic manner and the parsing algorithm from upper-layer protocols can execute in a completely automatic manner. One might view the results obtained by our parsing algorithms as an important indicator of penetration testing on in-vehicle CAN network. The results are also conducive to subsequent firmware parsing task and scientific research work that requires vehicle private protocols, e.g., IDS/IDPS.

As a countermeasure against our parsing algorithms, the vehicle manufacturers could perform lightweight encryption on the raw data to eliminate state dependencies and ensure the security of the firmware. They can also restrict the application scenarios of the upper-layer protocols. These mechanisms are related to the electrical electronic architecture of the vehicle systems.

## Figures and Tables

**Table 1:** CAN-TP header.

	Bit Offset	7–4 (Byte 0)	3–0 (Byte 0)	15–8 (Byte 1)	23–16 (Byte 2)	…
Frame						
Single		0	size	Data A	Data B	Data C
First		1	size		Data A	Data B
Consecutive		2	index	Data A	Data B	Data C
Flow control		3	FC flag	Block size	ST	/

**Table 2:** Results of the accelerator pedal position.

	Number of Points	20	50	100	200
Position					
0~$\frac{1}{3}$		0 × 488 4th byte (1.03)	0 × 488 4th byte (3.50)	0 × 488 4th byte (19.58)	0 × 488 4th byte (22.49)
0~$\frac{2}{3}$		0 × 488 4th byte (3.44)	0 × 488 4th byte (10.98)	0 × 488 4th byte (32.63)	0 × 488 4th byte (22.8)
0~1		0 × 488 4th byte (3.46)	0 × 488 4th byte (10.59)	0 × 488 4th byte (24.01)	0 × 488 4th byte (19.64)

**Table 3:** The percentage of parsing units removed by the filter conditions.

The First Stage	The Second Stage	Alternating Times
20.7%	0%	79.3%

## Data Availability

Not applicable.
